# Comparison of the *in vitro *and *in vivo *susceptibilities of *Burkholderia mallei *to Ceftazidime and Levofloxacin

**DOI:** 10.1186/1471-2180-9-88

**Published:** 2009-05-09

**Authors:** Barbara M Judy, Gregory C Whitlock, Alfredo G Torres, D Mark Estes

**Affiliations:** 1 Department of Pediatrics, University of Texas Medical Branch, Galveston, Texas, USA; 2 Department of Microbiology and Immunology, University of Texas Medical Branch, Galveston, Texas, USA; 3 Department of Clinical Laboratory Sciences, University of Texas Medical Branch, Galveston, Texas, USA; 4 Department of Pathology, University of Texas Medical Branch, Galveston, Texas, USA; 5 Sealy Center for Vaccine Development, University of Texas Medical Branch, Galveston, Texas

## Abstract

**Background:**

*Burkholderia mallei *is a zoonotic Gram negative bacterium which primarily infects solipeds but can cause lethal disease in humans if left untreated. The effect of two antibiotics with different modes of action on *Burkholderia mallei *strain ATCC23344 was investigated by using *in vitro *and *in vivo *studies.

**Results:**

Determination of minimal inhibitory concentrations (MICs) *in vitro *was done by the agar diffusion method and the dilution method. The MICs of levofloxacin and ceftazidime were in the similar range, 2.5 and 5.0 μg/ml, respectively. Intracellular susceptibility of the bacterium to these two antibiotics in J774A.1 mouse macrophages *in vitro *was also investigated. Macrophages treated with antibiotics demonstrated uptake of the drugs and reduced bacterial loads *in vitro*. The efficacy of ceftazidime and levofloxacin were studied in BALB/c mice as post-exposure treatment following intranasal *B. mallei *infection. Intranasal infection with 5 × 10^5 ^CFUs of *B. mallei *resulted in 90% death in non-treated control mice. Antibiotic treatments 10 days post-infection proved to be effective *in vivo *with all antibiotic treated mice surviving to day 34 post-infection. The antibiotics did not result in complete clearance of the bacterial infection and presence of the bacteria was found in lungs and spleens of the survivors, although bacterial burden recovered from levofloxacin treated animals appeared reduced compared to ceftazidime.

**Conclusion:**

Both antibiotics demonstrated utility for the treatment of glanders, including the ability for intracellular penetration and clearance of organisms *in vitro*.

## Background

*Burkholderia mallei*, the causative agent of glanders, a primary equine disease, is a Gram-negative, facultative intracellular bacterium which can be transmitted to humans with fatal consequences [1]. Human infections typically occur in people who have direct contact with glanderous animals such as veterinarians, farmers or laboratory workers. The most likely route of transmission involves contact of infectious exudates with cuts and abrasions or with mucosal membranes. *B. mallei *are also highly infectious organisms by aerosol and it is widely believed that it harbors the potential for use as a biological weapon [2]. In fact, the bacterium was one of the first agents used in biologic warfare during the American Civil War, World Wars I and II, and Russian invasion of Afghanistan. Consequently, it has been placed on the CDC category B agent list [3]. Inhalation of aerosol or dust containing *B. mallei *can lead to septicemia, pulmonary or chronic infections of the muscle, liver and spleen. The disease has a 95% case fatality rate for untreated septicemia infections and a 50% case fatality rate in antibiotic-treated individuals [4]. The ability of *B. mallei *to cause severe, rapidly fatal invasive infection initiated via aerosol in animals and humans, coupled with intrinsic resistance to antibiotics and diagnostic difficulty at early stage of disease make the bacterium a good candidate as a possible biological threat agent [5,6]. Our knowledge of pathogenesis of disease due to *B. mallei *is minimal. The disease was eliminated from domestic animals in the United States during the 1940s and the last reported naturally acquired human case in the United States occurred in 1945. There is little data available on antibiotic treatment of glanders and human cases are treated with the same regimens used for melioidosis, an endemic disease in Southeast of Asia and Northern Australia, caused by *Burkholderia pseudomallei*. Only one case of laboratory-acquired human glanders was reported to CDC recently [7]. This single human case of glanders corroborated *in vitro *data with *in vivo *efficacy for the *B. mallei *ATCC 23344 strain when a combination of intravenous doxycycline plus imipenem followed by oral doxycycline plus azithromycin successfully controlled a disseminated infection [7]. However, at present, the treatment of *B. mallei *with antibiotic therapy is still not well established and no effective vaccines are available.

Few *in vitro *antibiotic susceptibility studies for *B. mallei *have been performed. The antibiotic susceptibility of *B. mallei *is similar to that of *B. pseudomallei*, with resistance to a number of antibiotics [8]. Both organisms appear to be sensitive to imipenem and doxycycline, while most strains are susceptible to ceftazidime, ciprofloxacin, and piperacilin [9]. Unfortunately clinical experience with *B. pseudomallei *infections has shown that despite good *in vitro *activity, an antibiotic may be ineffective *in vivo *[10,11]. We chose ceftazidime, highly recommended drug for treatment of melioidosis. Ceftazidime belongs to the beta-lactam group, a broad spectrum antibiotic, structurally and pharmacologically related to penicillins, which work by inhibiting the bacterial cell wall synthesis. This third generation cephalosporin is effective against *Pseudomonas *and other Gram-negative bacteria. The second antibiotic chosen, levofloxacin, belongs to the quinolone group which inhibits the bacterial DNA gyrase in Gram-negative bacteria, thereby inhibiting DNA replication and transcription. Quinolones can enter cells easily and therefore are often used to treat intracellular pathogens. As there is a need for effective treatment and post-exposure prophylaxis, the objective of this study was to assess the *in vitro *susceptibilities of these antibiotics with different modes of action and compare with efficacy in macrophages and mice infected with *B. mallei*.

## Results

### Susceptibility testing, MIC determination

MICs were determined by the agar diffusion method and dilution method. The results from the agar diffusion method are listed in Tables 1 and 2. Our results indicate that *B. mallei *strain ATCC 23344 is susceptible to a concentration as low as 10 μg/ml of ceftazidime and 25 μg/ml of levofloxacin comparable to our *E. coli *control strain. The MICs were further evaluated by the dilution method for confirmation, resulting in 5 μg/ml of ceftazidime or 2.5 μg/ml of levofloxacin sufficient to inhibit the growth of *B. mallei *in LBG after 18–24 h incubation at 37°C under shaking conditions.

**Table 1:** Inhibition zone size standards for *B. mallei *for ceftazidime disks

Disk potency (mg/ml)	Zone diameter (mm) for *B. mallei *ATCC23344	Pattern of resistance/suceptibility
10	> 32	Susceptible
1	> 32	Susceptible
1 × 10^-1^	32	Susceptible
1 × 10^-2^	30	Susceptible
1 × 10^-3^	19	Intermediate
1 × 10^-4^	< 1	Resistant
1 × 10^-5^	< 1	Resistant
1 × 10^-6^	< 1	Resistant

**Table 2:** Inhibition zone size standards for *B. mallei *for levofloxacin disks

Disk potency (mg/ml)	Zone diameter (mm) for *B. mallei *ATCC23344	Pattern of resistance/susceptibility
2.5	> 40	Susceptible
2.5 × 10^-1^	> 40	Susceptible
2.5 × 10^-2^	27	Susceptible
2.5 × 10^-3^	10	Intermediatee
2.5 × 10^-4^	< 1	Resistant
2.5 × 10^-5^	< 1	Resistant
2.5 × 10^-6^	< 1	Resistant
2.5 × 10^-7^	< 1	Resistant

### *In vivo *post-exposure prophylaxis with levofloxacin and ceftazidime

**Figure 1:**
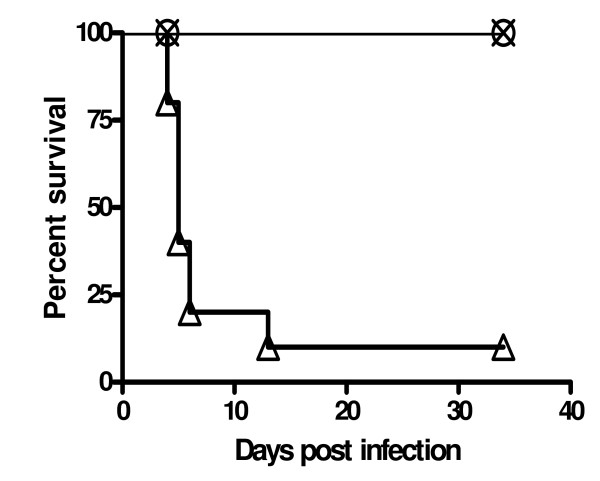
**Percentage of survival of BALB/c mice challenged with 5 × 10^5 ^CFUs of *B. mallei *intranasally (n = 10)**. Treatment with antibiotic started 24 hours post-infection, once a day, for 10 days. Ceftazidime (**X**) and levofloxacin (○) were administrated i.p. in doses 100 mg/kg/day and 20 mg/kg/day respectively. The infection of *B. mallei *resulted in 90% death in non-treated animals (△). All antibiotic treated mice survived to day 34 post-infection. Experiment performed twice, P < 0.0001 for non-treated vs. antibiotic treated animals.

The confirmed challenge dose of *B. mallei *was 4.7 × 10^5 ^CFU per animal delivered i.n. in 50 μl PBS (25 μl per nare). Non-treated control animals became sick within 48 h post-challenge indicated by non-specific signs such as piloerection and hypo-activity with trembling. The infection progressed with first deaths observed by day 4 post-challenge (Fig. 1). By day 6, 80% of non-treated control animals were dead with only one survivor in this group by day 34 (which lacked severe signs consistent with disease). Ceftazidime and levofloxacin, administrated i.p. 24 hours post-challenge, once a day, for 10 days, significantly reduced signs of the disease and proved to be effective with 100% survival rates at day 34 (P < 0.0001) on both treatments. Histological examination of organs from antibiotic treated survivors showed highly enlarged spleens with large, multifocal abscesses with extension into abdominal muscles in all infected animals (data not shown). The spleens of both antibiotic treated and non-treated animals exhibited a 6–10 fold increase in weight compared to uninfected healthy animals. Both spleens and livers showed myeloid hyperplasia. Interestingly, no lesions were found in the lungs of animals (data not shown).

### Bacterial load at day 34 post-infection

**Figure 2:**
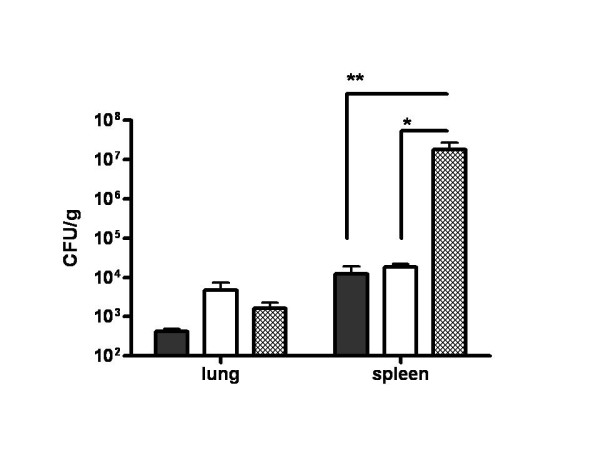
**Reduced *B. mallei *bacterial burden in antibiotic treated BALB/c mice**. Thirty-four days post-challenge, surviving levofloxacin treated mice (black bars), ceftazidime treated mice (white bars) and untreated control mice (crossed bars) were euthanized, and lungs and spleens were harvested, weighed and serial dilutions plated for CFU/g tissue weight., * P < 0.05, ** P < 0.01. Errors bars represent mean ± SEM.

Harvested lungs and spleens from each group of animals challenged with 5 × 10^5 ^CFU/50 μl by i.n. route were subjected to plating on LBG for CFU determination per gram of organ weight. One animal from levofloxacin treatment was free of bacteria in spleen and liver. The spleen from this animal looked normal, was not enlarged, suggesting that in this particular case, infection was not effective. Bacterial counts in the spleens from remaining antibiotic treated animals were similar, 1.9 × 10^4 ^± 3.9 × 10^3 ^CFU/g for ceftazidime and 1.2 × 10^4 ^± 6.6 × 10^3 ^CFU/g for levofloxacin and significantly lower from non-treated control animals (1.8 × 10^7 ^± 8.6 × 10^6 ^CFU/g of spleen, Fig. 2). By day 34 post-infection, bacteria was largely cleared from the lungs with no significant differences between antibiotic treated and non-treated animals, although bacterial burden of the spleens suggested that all animals developed chronic infection with *B. mallei*.

### The efficacy of ceftazidime and levofloxacin to kill intracellular bacteria *in vitro*

**Figure 3:**
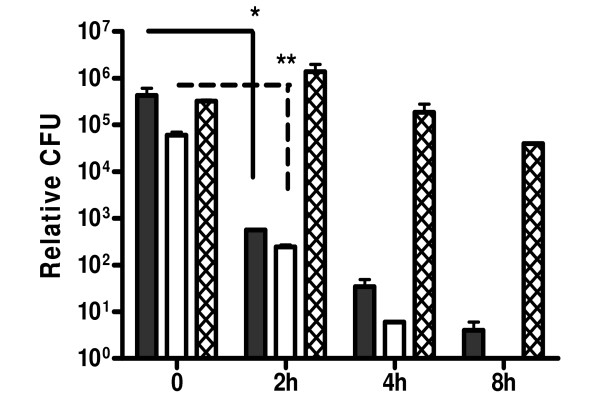
**Antibiotic mediated intracellular killing of *B. mallei *infected J774A.1 murine macrophages**. Bacteria were added at an MOI of 25:1 and incubated for 2 hours at 37°C with 5% CO_2 _followed by incubation with 100 × MIC levofloxacin (black bars), ceftazidime (white bars) or media only (crossed bars). Media in control wells contained 250 μg/ml kanamycin for first 2 h postinfection and 100 μg/ml kanamycin for the rest of the assay to prevent the growth of extracellular bacteria. At 2, 4 and 8 h post treatment, cells were washed and lysed with 0.1% Triton X-100, followed by serial 10-fold dilutions plated on LBG plates and incubated at 37°C for 2 days for CFUs determination. Experiment performed twice in triplicate. Errors bars represent mean ± SEM. * P < 0.05 significant difference between time 0 and all time points in levofloxacin treatment, ** P < 0.01 significant difference between time 0 and all time points in ceftazidime treatment.

For the determination of intracellular killing of *B. mallei *by antibiotics of interest, we performed a bacterial uptake assay by murine macrophages J774A.1 and evaluated bacterial killing for 8 hours of continuous exposure to antibiotics in concentrations equal to 100 × MIC for each compound tested. Murine J774A.1 cells were infected at an MOI of 25:1 and incubated for 2 hours in the absence of any antibiotics to allow for uptake (Time 0). At two hour intervals post-antibiotic exposure, intracellular CFU were determined resulting in a significant reduction of intracellular bacteria which continued throughout the assay (Fig. 3). Media in control wells contained 250 μg/ml kanamycin for first 2 h postinfection and 100 μg/ml kanamycin for the rest of the assay to prevent the growth of extracellular bacteria[12,13]. Media was free of bacteria throughout the entire experiment, suggesting efficient killing of extracellular bacteria (data not shown). At the end of experiment, after 8 hours post-exposure to antibiotics, intracellular *B. mallei *CFUs were negligible from cell lysates. Similar results were obtained with lower antibiotics concentration 10 × MIC and lower MOI, 12:1 (data not shown). The lactate dehydrogenase (LDH) cytotoxicity assay was performed during bacterial invasion assays to monitor cytotoxic effects of bacteria on J774A.1 macrophages. Throughout the assay LDH levels were below 20%. Cytotoxicity was observed at 8 h in ceftazidime treated macrophages, reaching 25.7% which may have contributed to the decrease in recoverable intracellular bacteria in this treatment. Possible cytotoxic effects of antibiotics alone was tested in separate experiments for up to 24 h, including concentrations higher than that tested, showing no significant LDH levels (data not shown).

## Discussion

Limited data of *in vitro *antibiotic susceptibilities to strains of *B. mallei *has been published. The recommendations for treatments of glanders are largely based on knowledge of pathogenesis of melioidosis, a human disease caused by a closely related species *B. pseudomallei*. Currently, ceftazidime is the first antibiotic of choice for treatment of acute melioidosis [14]. The previously established MICs of 16 different antimicrobials evaluated against both species showed most strains susceptible to ceftazidime, ciprofloxacin, imipenem, and doxycycline [8]. Although *B. mallei *has a susceptibility profile similar to *B. pseudomallei*, the MICs are usually lower in case of *B. mallei *[15]. Due to emergence of resistant strains and cases of disparity between *in vitro *susceptibility and clinical outcome of the treatments for melioidosis, the development of effective treatments has been difficult [10,16,17]. Both species, *B. mallei *and *B. pseudomallei*, share morphological, biochemical and antigenic characteristics, and could be expected that similar problems will occur in the case of *B. mallei*. There is a need for an extensive evaluation of susceptibility of antibiotics to these pathogens beyond *in vitro *studies.

Animal models to study equine glanders have been established [18] while there is a general lack of infection models that mimic human infection. Among rodents, guinea pigs and hamsters are most susceptible to glanders [19]. Mice, on the other hand, have similar resistance to glanders infections as humans, which makes this model more suitable to study therapies for *B. mallei*. Only intraperitoneal pathogenesis of glanders has been well described in the mouse model [20] with more recent studies of the bacterium administered via the aerosol or intranasal routes [21].

Here, we evaluated the susceptibilities *in vitro *of *B. mallei *to ceftazidime and levofloxacin, and their efficacy *in vivo *using intranasal infection in BALB/c mice, as inhalation would be the most likely route of infection in the event of bioterrorism threat. In previous *in vitro *studies, ceftazidime proved to be effective against *B. mallei *among others including imipenem, doxycycline, piperacillin, ciprofloxacin [8,9]. Levofloxacin demonstrates relatively high levels of activity against *B. mallei *but not *B. pseudomallei *[22]. Levofloxacin is known to achieve higher intracellular concentration and is recommended for intracellular infections [23]. Our results indicate that *B. mallei *strain ATCC 23344 is susceptible to a concentration as low as 2.5 μg/ml of levofloxacin and 5 μg/ml of ceftazidime. These results confirmed prior studies evaluating susceptibility of 15 isolates of *B. mallei *to 35 antimicrobial agents [15]. In this study, ceftazidime and levofloxacin appeared in the group of most effective drugs tested in this panel against *B. mallei*. However, the high percentage of resistant strains of *B. pseudomallei *to levofloxacin and the emergence of ceftazidime-resistant clinical isolates of *B. pseudomallei *would affect the recommendations of these drugs as useful treatment for both glanders and melioidosis, underlining the need for supplementary monitoring of the effectiveness of the recommended antimicrobials.

The effectiveness of levofloxacin and ceftazidime *in vitro *were substantiated in our *in vivo *experiments with all treated mice surviving at least 34 days post infection. The intranasal infection of mice with 5 × 10^5 ^CFUs of *B. mallei *resulted in 90% death in untreated control mice. Treatment with antibiotics used in this study prevented the development of an acute lethal form of disease but lacked the ability to provide complete clearance of the bacterial infection. By 34 days post-infection, bacteria were largely cleared from the lungs with no significant differences between treatments. Interestingly, in our intranasal infection model, the spleen appears to be the major target tissue for glanders infection and a site of multifocal abscesses. Similar findings were documented in studies with intraperitoneal glanders in mice and hamsters [20,24]. The untreated and antibiotic-treated mice exhibited a 6–10 fold increase in spleen weights compared to healthy, uninfected animals. Bacterial loads in spleens were significantly reduced in antibiotic treated animals compared to untreated control but remained in the range of 1.6 × 10^4 ^CFU/g of spleen. The antibiotics administrated 24 hours post-infection for 10 days led to the development of a chronic, non-lethal abscess infection suggesting that *B. mallei *may have the propensity for latency, as does the very closely related organism *B. pseudomallei *[25]. Efficacy of other antibiotics tested in hamsters revealed that time of administration of antimicrobials is the important factor affecting protection against *B. mallei *[24]. The experiments showed that administration of treatment less than 24 h post-exposure resulted in protection against the pathogen. A similar conclusion was obtained in antibiotics efficacy testing against *B. pseudomallei *infected mice [26]. Combined, this suggests that the infection could be contained or eliminated if very early antibiotic treatment was initiated to prevent the bacterial load from reaching a lethal dose in the host. The pharmacokinetics of each antimicrobial, relative to the *in vitro *MIC and the ability of the bacteria to reside in privileged intracellular sites (not always easily accessible to the antimicrobials) should be considered as an important factor in effective treatment. For that reason, we tested levofloxacin in our study since fluoroquinolones are known to penetrate renal, lung and bronchial track tissues achieving a high intracellular concentration exceeding levels of the drug in serum [23]. Both antimicrobials were very effective in intracellular bacterial killing reducing bacterial loads to practically undetectable levels, validating their ability as cell-permeable antibiotics.

## Conclusion

The current study showed that both ceftazidime and levofloxacin, despite good activity *in vitro *against *B. mallei*, failed to eradicate bacterium and resulted in development of a chronic, non-lethal form of glanders. Both antibiotics demonstrated some utility for treatment of glanders, including the ability for intracellular penetration and clearance of organisms *in vitro*, despite bacterial burdens recovered *in vivo *following i.p. antibiotic treatment.

## Methods

### Bacterial strain

*B. mallei *strain ATCC 23344 (China 7) was cultured on Luria-Bertani supplemented with 4% glycerol (LBG) agar plates for 48 h at 37°C. Isolated colonies were sub-cultured to LBG broth, and cultures were incubated at 37°C until optical density readings at 600 nm (OD_600_) reached an exponential phase of growth. Bacteria were pelleted by centrifugation, washed and re-suspended in sterile 1× phosphate-buffered saline (PBS, pH 7.4) to obtain the desired CFU/ml. All procedures were performed in a biosafety level 3 laboratory.

### Antimicrobial susceptibility testing (MIC determination)

The minimal inhibitory concentrations of the antibiotics were tested by the Kirby-Bauer disk diffusion method and the dilution method as previously described (Clinical and Laboratory Standards Institute, formerly NCCLS. 20003. Performance standards for antimicrobial disk susceptibility tests. 309 Approved standard – Eighth Edition M2-A8, ISBN 1-56238-485-6, CLSI. Wayne, Pa.). Briefly, fresh antibiotic-containing disks (serial dilutions) were used for susceptibility testing. LBG plates were inoculated with *B. mallei *ATCC 23344 and disks containing the antibiotic dilutions placed on top of the inoculated agar. The plates were incubated at 37°C for 24–48 h. Zones were measured and the mean diameter was calculated. The interpretation of results was based on the NCCLS zone diameters used for non-*Enterobacteriaceae*. For the broth dilution method, an inoculum of 10^5 ^CFU of washed *B. mallei *per ml was used, and the test was conducted in LBG for 24 h at 37°C. The interpretation of results was based on the NCCLS MIC breakpoints for non-*Enterobacteriaceae *and MIC for *B. pseudomallei *[16]. The inhibition of growth was confirmed by spectrophotometrically measurements and plating of serial dilutions onto LBG plates. Tubes containing bacteria but not antibiotic were included as a positive growth control.

### Mice

Animal studies were carried out in accordance with the Animal Care and Use Committee's guidelines as recommended by the National Institutes of Health. Female, 6- to 8-week-old, BALB/c mice were obtained from Harlan Sprague Dawley, Inc. (Indianapolis, Indiana). Animals were provided with rodent feed and water ad libitum and maintained on 12 h light cycle.

### Challenge with *B. mallei *and antimicrobial administration

Groups of 10 animals were inoculated via intranasal (i.n.) route with 5 × 10^5 ^CFUs of *B. mallei *ATCC 23344, in a total volume of 50 μl in PBS solution given to both nares. Treatment with antibiotic via intraperitoneal route (i.p.) started 24 hours after infection, once a day, for 10 days. Doses of antibiotics used in this study were in the range of high doses used in humans: ceftazidime 100 mg/kg/day and levofloxacin 20 mg/kg/day. The animals were weighed prior to challenge and doses of antibiotics were adjusted accordingly. Levofloxacin (Levaquin Injection, GlaxoSmithKline) and ceftazidime (Fortaz, Ortho-McNeil, Inc.) were purchased through local UTMB Pharmacy and doses for injection were prepared and stored according to manufacturer's instructions.

### Bacterial load determinations

Five animals from each group of antibiotic treated animals and survivors from non-treated control animals, were sacrificed and lungs and spleen were harvested for CFU determination. Organs were weighed, homogenized in 5 ml sterile PBS, plated in duplicates on LBG and incubated at 37°C for 2 days prior to CFU determinations. For comparison, spleen weights from healthy non infected but antibiotic treated animals were also evaluated. CFU were expressed as the mean ± SEM. Organs (lung, spleen and liver) from additional remaining survivors were used for histological examinations.

### *B. mallei *J774A.1 uptake and killing assays

Murine J774A.1 cells were seeded (5 × 10^5^) onto Corning Costar 24 well plates (Corning, NY) with DMEM and incubated overnight at 37°C with 5% CO_2_. Bacteria were added at an MOI of 25:1 to J774A.1 cells in duplicate. The high MOI was used to guaranty that every macrophage was able to take up a large number of bacteria that survived the phagocytic activity of the cell but were killed by our experimental antibiotic treatment. Inoculated wells were centrifuged at 800 × g for 2 minutes and incubated for 2 hours at 37°C with 5% CO_2 _followed by a PBS wash (×3) and 2, 4 and 8 hours incubation with antibiotics. Media in control wells contained 250 μg/ml kanamycin for first 2 h postinfection and 100 μg/ml kanamycin for the rest of the assay to prevent the growth of extracellular bacteria [12,13]. The concentration of antibiotics tested in this assay was equal to 100 × MIC for each compound. At appropriate time after incubation, cells were washed twice with PBS and lysed with 0.1% Triton X-100, followed by 10-fold serial dilutions plated on LBG plates and incubated at 37°C for 2 days prior to colony forming units determination. Additionally, to monitor the J774A.1 cells during experiment, LDH (lactate dehydrogenase) cytotoxicity assay was performed according to manufacturer's instruction (BioVision Research Products, Mountain View, CA) at all time points.

### Statistical analysis

Survival curves were calculated by Kaplan Meier survival analysis with log-rank tests between groups using GraphPad Prism (V.4.03 for windows). Comparisons of spleen weights were performed using ANOVA and LOG transformed values of bacterial load was analyzed by Student's t-test. P value ≤ 0.05 was considered significant.

## Abbreviations

MICs: Minimal inhibitory concentrations; i.p.: intraperitoneal; i.n.: intranasal.

## Authors' contributions

BMJ designed and conducted experiments and drafted the manuscript. GCW contributed to design and conduct of experiments and drafting manuscript, AGT conducted and provided analysis of the bacterial work, DME conceived the study, participated in its design and coordination and helped to draft the manuscript. All authors read and approved the final manuscript.
